# Uterine and ovarian carcinosarcomas overexpressing Trop-2 are sensitive to hRS7, a humanized anti-Trop-2 antibody

**DOI:** 10.1186/1756-9966-30-106

**Published:** 2011-11-10

**Authors:** Rhoda Raji, Federica Guzzo, Luisa Carrara, Joyce Varughese, Emiliano Cocco, Stefania Bellone, Marta Betti, Paola Todeschini, Sara Gasparrini, Elena Ratner, Dan-Arin Silasi, Masoud Azodi, Peter Schwartz, Thomas J Rutherford, Natalia Buza, Sergio Pecorelli, Alessandro D Santin

**Affiliations:** 1 Department of Obstetrics, Gynecology & Reproductive Sciences, Yale University School of Medicine, 333 Cedar Street, New Haven, CT, USA; 2 Department of Pathology, Yale University School of Medicine, 333 Cedar Street, New Haven, CT, USA; 3 Division of Gynecologic Oncology, University of Brescia, Brescia, Italy

**Keywords:** carcinosarcoma, hRS7, immunotherapy, natural killer cell, Trop-2

## Abstract

**Background:**

We evaluated the expression of human trophoblastic cell-surface marker (Trop-2) and the potential of hRS7 - a humanized monoclonal anti-Trop-2 antibody - as a therapeutic strategy against treatment-refractory human uterine (UMMT) and ovarian (OMMT) carcinosarcoma cell lines.

**Materials and methods:**

Trop-2 expression was evaluated by immunohistochemistry (IHC) in paraffin-embedded tumor tissues, by real-time polymerase-chain-reaction (RT-PCR) and flow-cytometry in cell lines. Sensitivity to hRS7 antibody-dependent cellular cytotoxicity (ADCC) and complement-dependent cytotoxicity was tested using 5-hour chromium-release assays against UMMT and OMMT cells.

**Results:**

Trop-2 expression was elevated in 9 of 26 (35%) UMMT and 8 of 14 (57%) OMMT tissues tested by IHC. Positivity for Trop-2 mRNA by RT-PCR and surface expression by flow cytometry were detected in 2 of 4 cell lines, with high positivity noted in OMMT-ARK-2. OMMT-ARK-2 was highly sensitive to hRS7 ADCC (range: 34.7-41.0%; *P *< 0.001) with negligible cytotoxicity seen in the absence of hRS7 or in the presence of control antibody (range: 1.1-2.5%). Human IgG did not significantly inhibit ADCC while human complement increased, hRS7-mediated-cytotoxicity against OMMT-ARK-2.

**Conclusion:**

Trop-2 is overexpressed in a proportion of UMMT and OMMT, and hRS7 may represent a novel, potentially highly effective treatment option for patients with treatment-refractory carcinosarcomas overexpressing Trop-2.

## Background

Carcinosarcomas, also known as Mixed Mullerian Tumors (MMT), of the female genital tract are rare tumors that most commonly arise in the uterus, followed by the ovaries, fallopian tubes, and the vagina [1]. The pathogenesis of carcinosarcomas remains under debate, but an increasing body of evidence supports the origin of both elements from a common epithelial cell line that undergoes sarcomatous dedifferentiation, rather than two independent progenitors [2]. Carcinosarcomas are histologically comprised of malignant epithelial and mesenchymal components and may be classified based on the nature of their mesenchymal elements. Tumors with "homologous" mesenchymal components differentiate towards tissues physiologically native to the primary site (e.g. leiomyosarcoma component), while heterologous tumors contain mesenchymal components that are physiologically foreign to the primary site (e.g. chondrosarcoma component).

Uterine cancer is the most prevalent gynecologic malignancy and the 4^th ^most prevalent cancer among United States women, with an estimated 43,470 new cases and 7,950 cancer-related deaths in 2010 [3]. Carcinosarcomas comprise 2-5% of all uterine malignancies and have an estimated recurrence rate of 40-60% [4], with approximately 35% of patients having extra-uterine disease at diagnosis. Although surgical debulking is the mainstay of treatment, the high rate of tumor recurrence and a poor median survival of 16-40 months after diagnosis suggest a need for improved adjuvant treatment [5,6].

Cancer of the ovary is the 9^th ^most common malignancy and the 5^th ^leading cause of cancer-related death among U.S. women, with an estimated 28,880 new cases and 13,850 associated deaths in 2010 [3]. Carcinosarcomas comprise less than 1-2% of these tumors [4], with most individuals having nodal metastases at diagnosis and 75% of women being found to have stage III or IV disease at surgical staging. Ovarian carcinosarcoma portends a worse prognosis than uterine carcinosarcoma, with a median survival rate of 8-32 months and recurrence rates of 50-100% [4,6]. Cytoreductive surgery followed by combination platinum-based chemotherapy appears to confer the best survival benefits, with attendant notable morbidity risks and continued dismal long-term survival data.

There is a clear need to better understand the molecular basis of carcinosarcomas and to develop more effective treatment modalities against these aggressive tumors. Trop-2 (also referred to as EGP-1, TACSTD2, M1S1, and GA733-1) is a monomeric transmembrane cell surface glycoprotein that was originally identified in human placental trophoblastic tissue. It is expressed by several human epithelial cancers but has limited expression in normal human cells [7]. Little is known about the physiologic role of Trop-2 and the nature of its role as an oncogene remains unclear. Trop-2 has been implicated in activation of the ERK/MAPK pathway, leading to downstream alterations in cell proliferation, migration, invasion, and survival [8]. Clinically, Trop-2 overexpression has been associated with increased tumor invasiveness and decreased overall survival in multiple types of human carcinomas. Our group has previously identified Trop-2 overexpression in serous ovarian cancer and uterine serous papillary carcinoma (USPC), two notably aggressive, treatment-resistant gynecologic malignancies. We have also identified Trop-2 as an independent marker for decreased survival in patients with epithelial ovarian carcinomas [9,10].

The differential expression of Trop-2 in cancers compared to normal tissue, its association with clinically important tumor behavior, and its histologic accessibility as a transmembrane receptor make it an attractive target for immunotherapy. Importantly, a murine monoclonal antibody (mAb), mRS7, generated by hybridoma technology against Trop-2, has been shown to be effective as a radiolabeled, as well as drug- and toxin-conjugated, immunotherapeutic agent in xenograft cancer models [11-15]. In this study we aimed to investigate the potential of hRS7, a humanized anti-Trop-2 monoclonal antibody, in the treatment of uterine and ovarian carcinosarcomas.

## Materials and methods

### Trop-2 Immunostaining of Formalin-Fixed Tumor Tissues

Carcinosarcoma specimens (26 uterine and 14 ovarian), normal ovarian, and endometrial control tissues were evaluated by standard immunohistochemical staining (IHC) on formalin-fixed tumor tissues for Trop-2 surface expression. Patient characteristics from which tumor and normal samples were obtained are described in Table 1. IHC staining for Trop-2 were performed on 4-μm-thick sections of formalin-fixed, paraffin-embedded tissue with purified goat polyclonal antibody against the recombinant human Trop-2 extracellular domain (R&D Systems, Inc., Minneapolis, MN; diluted 1:100), as described previously [9].

**Table 1:** Patient Characteristics

Pathology and Tissue Type (number)	Age in Years		Race		Stage			
	
	Mean	(SD)	AA^1^	C^2^	I	II	III	IV
Formalin Fixed NEC^3^(5)	66	(4)	3	2				

Formalin Fixed NOVA^4 ^(3)	67	(6)	1	2				

Formalin Fixed UMMT and OMMT								

UMMT (26)	66	(9)	10	16	14	4	5	3

OMMT (14)	72	(7)	5	9	4	3	5	2

Carcinosarcoma cell lines								

Primary UMMT (2)	58	(12)	1	1	1	1		

Primary OMMT (2)	67	(9)	1	1		1		1

^1^AA - African-American

^2 ^C - Caucasian

^3^NEC - Normal Endometrial Cells

^4^NOVA - Normal Ovarian Cells

### Establishment of Carcinosarcoma Cell Lines

Study approval was obtained from the Institutional Review Board and informed consent was obtained from all patients, per institutional guidelines. Fresh, surgical tumor biopsies were collected and patients were staged according to the International Federation of Gynecologists and Obstetricians 1988 operative staging system. Two primary uterine carcinosarcoma cell lines (UMMT-ARK-1 and UMMT-ARK-2) and two primary ovarian carcinosarcoma cell lines (OMMT-ARK-1 and OMMT-ARK-2) were established after sterile processing of surgical specimens as previously described [9,10]. Briefly, tumor tissue was mechanically minced to portions no larger than 1 to 3 mm^3 ^in an enzyme solution made of 0.14% collagenase type I (Sigma) and 0.01% DNase (Sigma, 2000 KU/mg) in RPMI 1640, and incubated in the same solution in a magnetic stirring apparatus for an hour at room temperature. Enzymatically dissociated cells were then washed twice in RPMI 1640 with 10% fetal bovine serum and maintained in RPMI supplemented with 10% fetal bovine serum, 200 μg/ml of penicillin and 200 μg/ml of streptomycin at 37°C, 5% CO_2 _in 75 cm^2 ^tissue culture flasks or Petri dishes (Corning). After seeding on plasticware for 48-72 hours, nonadherent cells and contaminant inflammatory cells were gently removed from the culture by multiple washings with PBS. Both UMMTs were homologous and established from uterine biopsies of chemotherapy naïve patients at the time of staging surgery. UMMT-ARK-1 and UMMT-ARK-2 were established from patients harboring FIGO stage I and FIGO stage II disease, respectively. Of the OMMTs, one was homologous and one heterologous; both were obtained from metastatic sites in patients harboring recurrent, chemotherapy-resistant disease. These patients were initially diagnosed with FIGO stage II (OMMT-ARK-2) and FIGO stage IV (OMMT-ARK-1) ovarian cancer. Each cell line demonstrated high resistance to multiple chemotherapeutic agents including carboplatin, cisplatin, paclitaxel, doxorubicin, ifosfamide, gemcitabine and topotecan when tested *in vitro *by chemotherapy resistance assays (Extreme Drug Resistant (EDR) assay, Oncotech, Irvine, CA; Chemo Fx, Precision Therapeutics Inc., Pittsburgh, PA. Data not shown). Source-patient characteristics and initial staging data of these cell lines are described in Table 1.

### Quantitative Real-Time Polymerase Chain Reaction

RNA isolation from normal endometrium, ovarian epithelial control tissues and each primary carcinosarcoma cell line was performed using TRIzol Reagent (Invitrogen) following manufacturer instructions, as previously described [9]. Since Trop-2 is an intron-less gene, all RNA samples were treated with TURBO DNase enzyme (TURBO DNAfree Kit; Ambion, Inc., Applied Biosystem Business, CA) to remove contaminating DNA. Glyceraldehyde-3-phosphate dehydrogenase (GAPDH) Assay on Demand Hs99999905_m1 (Applied Biosystems, Foster City, CA) was an endogenous control used to normalize variations in cDNA quantities between samples. The qRT-PCR was performed in duplicate by using a primer set and probe specific for Trop-2 (ie, Trop2-EX56, forward: CGCCTTGGGTTTAAATTATTTGATGAGT; reverse: GCTACTACATAGGCCCAGTTAACAA). Quantitative real-time PCR (qRT-PCR) was performed with a 7500 Real-time PCR System per manufacturer protocols (Applied Biosystems) to evaluate Trop-2 expression in all samples. In brief, complementary DNA obtained from 50 ng of total RNA was amplified in a 25-μl PCR reaction following the manufacturer's recommended protocol and amplification steps: denaturation for 10 min at 95°C followed by 40 cycles of denaturation at 95°C for 15 s and annealing extension at 60°C for 1 min. The comparative threshold cycle (C_T_) method was used to determine gene expression in each sample relative to the value observed in a control cell line known to express Trop-2.

### Flow Cytometry

The humanized anti-Trop-2 monoclonal antibody, hRS7 (Immunomedics, Inc., Morris Plains, NJ), was used for flow cytometry studies. Each of the primary cell lines obtained from the patients described above was stained with 5 μg/mL of hRS7; similarly, 5 μg/mL of the chimeric anti-CD20 mAb rituximab (Rituxan, Genentech, San Francisco, CA) was used as a negative control. A goat anti-human F(ab)_2 _immunoglobulin (BioSource International, Camarillo, CA) was used as a secondary reagent. Analysis was conducted with FACScan, using Cell Quest software (Becton Dickinson, Franklin Lakes, NJ).

### Tests for Antibody Dependent Cell Cytotoxicity (ADCC)

A standard 5-hour chromium (^51^Cr) release assay was performed to measure the cytotoxic reactivity of Ficoll-PaqueTM PLUS (GE Healthcare, Uppsala, Sweden) separated peripheral blood lymphocytes (PBLs) obtained from several healthy donors against each cell line. The release of ^51^Cr from the target cells was measured as evidence of tumor cell lysis after exposure of tumor cells to 10 μg/mL of hRS7. Controls included the incubation of target cells alone as well as cells with PBLs alone or mAb alone. Rituximab was used as a negative control for hRS7 in all bioassays. ADCC was calculated as the percentage of killing of target cells observed with hRS7 plus effector cells compared with ^51^Cr release from target cells incubated alone.

### Test for Complement-Mediated Target Cell Lysis and Gamma (γ) -Globulin Inhibition

To evaluate the potential inhibition of ADCC against UMMT and OMMT cell lines by physiologic human plasma concentrations of **γ**-globulin, human plasma was added in the presence or absence of effector PBLs in a 1:2 ratio. This human plasma was used as a source of complement to test for complement-mediated target cell lysis. A standard 5 h ^51^Cr release assay was again used to assess the degree of cell lysis. In some experiments, heat-inactivated human plasma (56°C for 60 minutes) was added in the presence of effector PBLs. Controls included the incubation of target cells alone or with either lymphocytes or mAb separately. Rituximab was used as a control mAb.

### Statistical Analysis

For qRT-PCR data, the right skewing was removed by taking copy number ratios relative to the lowest-expressing normal endometrial cells (NEC) and normal ovarian sample (NOVA) (relative copy number), log2 transforming them to ΔCTs, and comparing the results by means of unequal-variance t-test for carcinosarcomas versus controls. Group means with 95% confidence intervals (CIs) were calculated by computing them on the ΔCTs and then reverse-transforming the results to obtain means (with 95% CIs) of mRNA relative expression. Differences in Trop-2 expression by flow cytometry were analyzed by unpaired *t*-tests, and a *P *value of < 0.05 between samples was considered to be significant. The Wilcoxon rank-sum (WRS) test was used to compare carcinosarcomas against controls for differences in IHC Trop-2 staining intensities. Sample-type differences were expressed as odds ratios accompanied by 95% confidence limits. Kruskal-Wallis test and chi-square analyses were used to evaluate differences in hRS7-induced ADCC levels in primary tumor cell lines. Statistical analysis was performed using PASW Version 18 (SPSS, Chicago, IL).

## Results

### Trop-2 Expression by Immunohistochemistry of Uterine and Ovarian Carcinosarcomas

**Figure 1:**
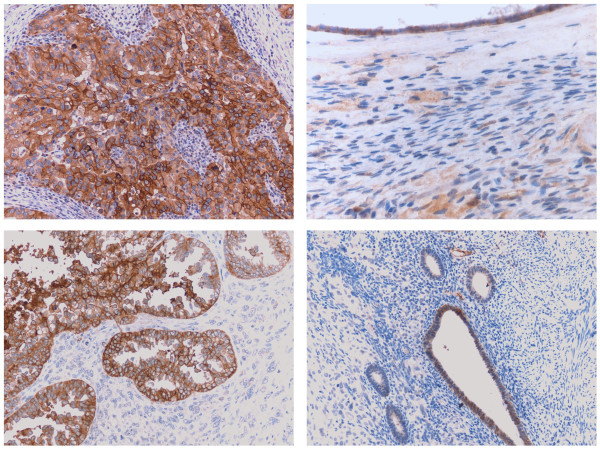
**Representative Trop2 immunostain in ovarian and uterine MMT and control normal tissues**. Upper left panel: Strong, diffuse membranous Trop2 expression (3+) in the carcinomatous component of ovarian MMT. Upper right panel: Minimal to absent Trop2 expression in normal ovarian surface epithelium and stroma. Lower left panel: Strong, focal membranous Trop2 expression (2+) in the carcinomatous component of uterine MMT. The adjacent sarcomatous component is negative for Trop2. Lower right panel: Weak, focal Trop2 expression in normal endometrial glands. (All images 200× original magnification)

We performed immunohistochemical analysis on formalin-fixed, paraffin-embedded tumor tissue from a set of 40 patients harboring uterine (UMMT, 26 patients) and ovarian (OMMT, 14 patients) carcinosarcomas. As representatively shown in Figure 1 and reported in Table 2, we found membranous positivity for Trop-2 in 9 of the 26 (35%) UMMT and 8 of the 14 (57%) OMMT samples tested. The intensity of Trop-2 staining was significantly higher among the tumor specimens compared with normal endometrial cells (Figure 1) and ovarian controls (WRS *P *≤ 0.005). Of the positive UMMT samples, 2 of the 9 specimens had a low positivity (1+) for Trop-2 protein, while the remaining specimens showed moderate (2+ in 4 samples) or strong (3+ in 3 samples) Trop-2 positivity. Among the positive OMMT samples, 2 of the 8 specimens showed low positivity (1+) for Trop-2 protein, while the remaining IHC specimens showed moderate (2+ in 3 samples) or strong (3+ in 3 samples) Trop-2 positivity. Without exception, Trop-2 positivity was detected only in the epithelial component of the carcinosarcoma specimens.

**Table 2:** IHC Results for Trop-2 Protein Expression in UMMT and OMMT

Patients	UMMT	OMMT
PT 1	3+	0

PT 2	0	3+

PT 3	2+	2+

PT 4	0	0

PT 5	0	0

PT 6	0	0

PT 7	0	1+

PT 8	0	0

PT 9	2+	0

PT 10	0	3+

PT 11	3+	1+

PT 12	0	3+

PT 13	0	2+

PT 14	0	2+

PT 15	1+	

PT 16	1+	

PT 17	0	

PT 18	3+	

PT 19	0	

PT 20	0	

PT 21	0	

PT 22	0	

PT 23	2+	

PT 24	0	

PT 25	2+	

PT 26	0	

### Trop-2 Transcript Levels in Carcinosarcomas

The ovarian and uterine carcinosarcoma cell lines were tested for Trop-2 expression by qRT-PCR. Table 1 shows the histopathologic characteristics of the patients from whom the cell lines were established. Trop-2 expression was significantly higher in two cell lines (UMMT-ARK-1 and OMMT-ARK-2) compared with normal control tissues (Table 3, *P *= 0.02). Among the carcinosarcomas tested, UMMT-ARK-1 demonstrated a low mRNA relative expression for Trop-2 (65.7) while OMMT-ARK-2 demonstrated a high mRNA relative expression for Trop-2 (13,032). Negligible Trop-2 expression by qRT-PCR was detected in the other cell lines, with UMMT-ARK-2 and OMMT-ARK-1 having 0.012 and 0.453 mRNA relative expression respectively (Table 3, *P *= 0.93).

**Table 3:** Trop-2 mRNA and Protein Expression in Carcinosarcoma Cell Lines

Cell Line	RT-PCR^1^	Flow cytometry	
**Cells (%)**	**MFI^2^**		

Control NEC^3^	1	-	-
Control NOVA^4^	1	-	-
UMMT ARK-1	65.7	100	20
UMMT ARK-2	0.5	0	0
OMMT ARK-1	0.1	0	0
OMMT ARK-2	13032	100	151

^1^RT-PCR - Real-time Polymerase Chain Reaction

^2 ^MFI - Mean Fluorescence Intensity

^3 ^NEC - Normal Endometrial Cells

^4 ^NOVA - Normal Ovarian Cells

### Trop-2 Surface Expression by Flow Cytometry in Primary Carcinosarcoma Cell Lines

To determine whether increased expression of Trop-2 mRNA corresponded with increased Trop-2 cell surface protein expression, we performed flow cytometry on all primary cell lines. Trop-2 surface expression by flow cytometry was found to significantly correlate with Trop-2 mRNA relative expression in all cell lines (Table 3). The difference in Trop-2 surface protein expression between cell lines with low/negligible Trop-2 expression and those with positive Trop-2 expression, as tested by flow cytometry, was statistically significant (*P *= 0.04) as well as mRNA relative expression between cell lines with low/negligible Trop-2 expression and those with positive Trop-2 expression (p < 0.05).

### Carcinosarcoma cell lines are sensitive to hRS7-mediated ADCC

**Figure 2:**
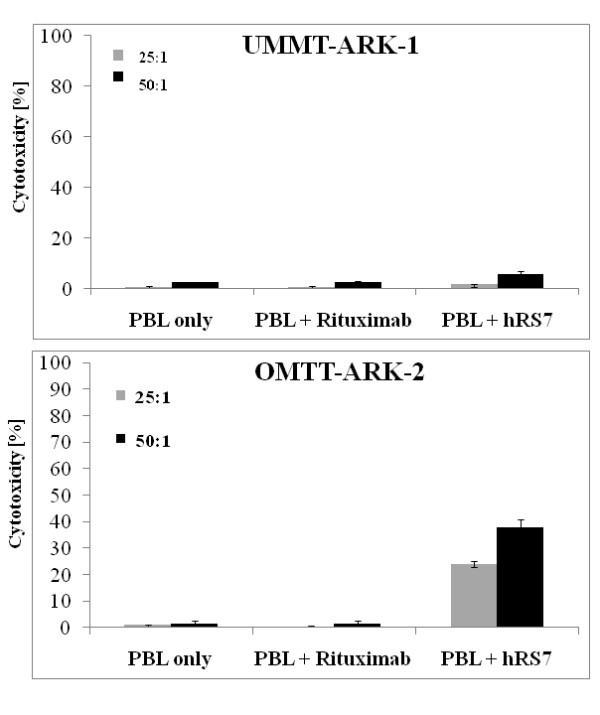
**Representative cytotoxicity experiments against the low Trop-2 expressing cell line**. UMMT-ARK-1 (2 a) and the high Trop-2 expressing cell line OMMT-ARK-2 (2 b) at different effector to target cell ratios in the presence or absence of hRS7 in a 5 h ^51^Cr-release cytotoxicity assay. Consistent with their Trop-2 expression levels, low degrees of ADCC was detected against UMMT-ARK-1 while a high degree of ADCC was detected against the OMMT-ARK-2 cell line. Negligible cytotoxicity was detectable in the absence of hRS7 or in the presence of rituximab control antibody against both cell lines.

Each carcinosarcoma cell line was tested for sensitivity to natural killer (NK) cell activity by exposure to peripheral blood lymphocytes (PBLs) collected from several healthy donors. Cytotoxicity was measured using a standard 5 h ^51^Cr-release assay. Without exception, all cell lines were found to be highly resistant to NK-mediated lysis when exposed to PBL with or without rituximab control antibody at effector: target cell ratios (E:T) of 25:1 and 50:1 (mean killing 0.9% ± 2.5 SD, Figure 2 and data not shown). Incubation with hRS7 resulted in a high degree of immune-mediated cell death in the Trop-2 overexpressing cell line (OMMT-ARK-2, mean of 37.7%, range of 34.7-41.0%; *P *< 0.001), while a low cytotoxic effect was detected against the low Trop-2 expressing cell line (UMMT-ARK-1, mean 5.7%, range: 4.4-6.7%; *P *= 0.02; Figure 2). Consistent with the negligible Trop-2 expression seen by qRT-PCR and flow cytometry (Table 3), UMMT-ARK-2 and OMMT-ARK-1 demonstrated no significant killing after incubation with PBL with hRS7 (data not shown).

### Effect of Complement and Physiologic Concentrations of IgG on hRS7-mediated ADCC

**Figure 3:**
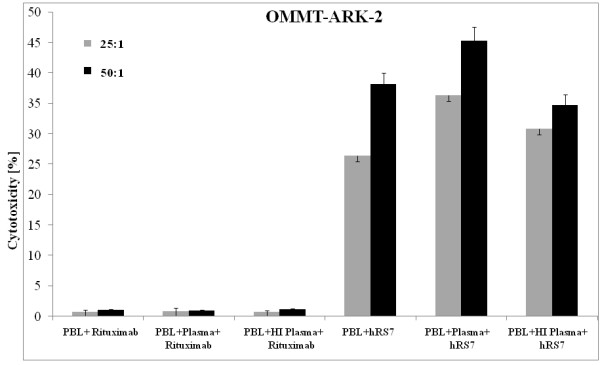
**Representative cytotoxicity experiments against the OMMT-ARK-2 cell line**. Cytotoxicity in the presence of human plasma diluted 1:2 (with or without heat-inactivation) with effector cells and either hRS7 or rituximab control antibody in 5 h ^51^Cr-release assays. Addition of untreated plasma (diluted 1:2) to PBL in the presence of hRS7 significantly increased the ADCC achieved in the presence of hRS7 and PBL against OMMT-ARK-2 (*P *= 0.002). Addition of physiological concentrations of IgG (i.e. heat-inactivated plasma diluted 1:2) to PBL in the presence of hRS7 did not significantly alter the degree of ADCC achieved against OMMT-ARK-2 in the presence of hRS7 and PBL (*P *= 0.95).

The OMMT-ARK-2 cell line was evaluated for sensitivity to complement-mediated cytotoxicity and for possible inhibition of ADCC by physiological concentrations of IgG. Human plasma (with or without heat inactivation) was added in the presence or absence of the effector cells and hRS7 in a 1:2 ratio, with the degree of cell lysis evaluated via 5 h ^51^Cr-release assays. Addition of plasma with or without hRS7 was unable to induce significant cytotoxicity against OMMT-ARK-2 cells in the absence of PBL (data not shown). However, incubation of plasma with PBL in the presence of hRS7 consistently increased hRS7-mediated cytotoxicity against OMMT-ARK-2 when compared to incubation with PBL alone (*P *= 0.002, Figure 3). Addition of physiologic concentrations of IgG (heat-inactivated plasma in a 1:2 ratio) to PBL in the presence of hRS7 did not significantly decrease hRS7-mediated killing (*P *= 0.95) when compared to incubation without plasma (Figure 3), suggesting that the presence of non-specific IgG does not alter the ability of hRS7 to mediate ADCC in Trop-2 expressing carcinosarcoma cells.

## Discussion

In this study, we have investigated Trop-2 expression and localization by immunohistochemistry in uterine and ovarian carcinosarcomas and compared these findings to normal endometrium and ovarian control tissues. We have evaluated Trop-2 expression in multiple biologically aggressive, chemotherapy-resistant carcinosarcoma cell lines. Additionally, we have tested the sensitivity of these primary cell lines to immune-mediated cell death in the presence of hRS7, a humanized Trop-2 mAb made by grafting the complementary-determining regions of its murine counterpart (mRS7) onto human IgG1 framework regions [11,13-15]. To our knowledge, this is the first time that Trop-2 protein has been demonstrated to be significantly upregulated in human carcinosarcomas from the uterus (UMMT) and ovary (OMMT), with negligible expression being detected in normal ovarian and uterine tissues. Significantly, Trop-2 positivity was confined to the epithelial component of the carcinosarcomas, without exception.

Although the relationship between high Trop-2 expression and the aggressiveness of human epithelial neoplasms remains unclear, there is evidence that Trop-2 functions in the transduction of cell signals regulating tumor cell growth and resistance to apoptosis. Trop-2 possesses cytoplasmic serine and tyrosine phosphorylation sites and might function as a cell signal transducer and regulator of tumor cell growth while increasing tumor cell resistance to apoptosis [16]. Consistent with this, Trop-2 has been identified as an oncogene, implicated in colon cancer tumor growth, migration, and invasion, which suggests that Trop-2- specific targeting may inhibit tumor cell growth, migration and invasion [17]. Several human cancers have been shown to express a bicistronic CYCLIN D1-TROP2 mRNA chimera that acts as an oncogene and is able to induce aggressive tumor growth [18]. These observations support the possibility that aberrant Trop-2 expression contributes to the enhanced biologic aggressiveness of multiple human cancers, including carcinosarcomas.

Importantly, in this study we have tested the capability of hRS7 to induce immune-mediated killing of primary uterine and ovarian carcinosarcoma cell lines that express increased levels of Trop-2. Two of the four cell lines available to this study, one uterine and one ovarian, were found have elevatedTrop-2 expression, with one cell line (OMMT-ARK-2) expressing high Trop-2 mRNA relative expression by PCR as well as high surface level Trop-2 protein expression by flow cytometry. This highly expressing cell line was found to have corresponding high sensitivity to hRS7-mediated ADCC, while negligible killing was detected in the presence of allogeneic PBL in the absence of hRS7 or in the presence of rituximab, used as a control antibody. These results suggest that uterine and ovarian carcinosarcomas, which are notoriously resistant to multiple clinically available chemotherapeutic agents [5,6], can be made highly sensitive to immune-mediated cytotoxicity when effector cells are engaged by the Trop-2-specific antibody, hRS7.

*In vivo*, ADCC applications are known to be dependent upon the availability of the effector cells (mainly natural killer cells) to interact with the antibody at the target site in the presence of high concentrations of irrelevant human IgG. In this study, we show that ADCC against carcinosarcomas was not significantly inhibited by high concentrations (up to 50%) of human plasma. In fact, a consistent increase in cytotoxicity was detected in the presence of effector cells and non-heat-inactivated human plasma. This suggests that in the presence of effector PBL, human plasma may augment hRS7-mediated cytotoxicity against carcinosarcomas. Moreover, these results indicate that the binding of hRS7 to the Fc receptor on mononuclear effector cells is likely to occur in the *in vivo *setting.

## Conclusions

In conclusion, this is the first report on Trop-2 protein expression and hRS7 antibody-dependent cellular cytotoxicity in uterine and ovarian carcinosarcomas. We report Trop-2 overexpression in 35% of uterine and 57% of the ovarian carcinosarcoma tested by IHC and in two out of four primary carcinosarcoma cell lines available to this study, and we have provided evidence that increased surface expression of Trop-2 is associated with increased cancer cell susceptibility to immune-mediated cytotoxicity in the presence of hRS7. Although *in vivo *data will ultimately be necessary to validate the therapeutic potential of hRS7 against Trop-2-expressing carcinosarcomas, our *in vitro *results suggest that targeting cancer cells with high surface expression of Trop-2 may be an effective way to treat residual or resistant uterine and ovarian carcinosarcomas.

## Competing interests

The authors declare that they have no competing interests.

## Authors' contributions

RR, FG, LC, SB, EC, MB, PT, SG, and JV carried out the molecular in vitro studies including RT-PCR, flow cytometry and IDCC assays, as well as statistical analysis. NB carried out the IHC studies on the tissue samples. DS, MA, PS, TR, SP, ER, and AS participated in the design of the study and drafted the manuscript. AS conceived the study. All authors read and approved the final manuscript.
